# Distributed Clone Detection in Static Wireless Sensor Networks: Random Walk with Network Division

**DOI:** 10.1371/journal.pone.0123069

**Published:** 2015-05-18

**Authors:** Wazir Zada Khan, Mohammed Y. Aalsalem, N. M. Saad

**Affiliations:** 1 Electrical and Electronic Engineering Department, Universiti Teknologi PETRONAS, Bandar Seri Iskandar, Tronoh, Perak Malaysia; 2 School of Computer Science & Information System, Jazan University, Jazan, Saudi Arabia; Tianjin University of Technology, CHINA

## Abstract

Wireless Sensor Networks (WSNs) are vulnerable to clone attacks or node replication attacks as they are deployed in hostile and unattended environments where they are deprived of physical protection, lacking physical tamper-resistance of sensor nodes. As a result, an adversary can easily capture and compromise sensor nodes and after replicating them, he inserts arbitrary number of clones/replicas into the network. If these clones are not efficiently detected, an adversary can be further capable to mount a wide variety of internal attacks which can emasculate the various protocols and sensor applications. Several solutions have been proposed in the literature to address the crucial problem of clone detection, which are not satisfactory as they suffer from some serious drawbacks. In this paper we propose a novel distributed solution called Random Walk with Network Division (RWND) for the detection of node replication attack in static WSNs which is based on claimer-reporter-witness framework and combines a simple random walk with network division. RWND detects clone(s) by following a claimer-reporter-witness framework and a random walk is employed within each area for the selection of witness nodes. Splitting the network into levels and areas makes clone detection more efficient and the high security of witness nodes is ensured with moderate communication and memory overheads. Our simulation results show that RWND outperforms the existing witness node based strategies with moderate communication and memory overheads.

## Introduction

Wireless Sensor Network (WSN) is a collection of sensor nodes with powerful sensing capabilities but limited resources. They consist of advanced network architectures and thus are used in a wide variety of applications [1][2]. These sensors lack tamper resistance hardware because of cost considerations and are often deployed in tough and rough settings and vicinities, hostile scenarios and unattended environments. Thus, they antagonize the extortions from the invaders and muggers which can launch many attacks including the intention to acquire critical information from the WSN or to debilitate and enervate the tasks of the WSNs. Here, we particularly focus on more harmful attack which is known as *node replication attack* or *clone attack*. In this attack an adversary physically captures one or more sensor nodes and compromise all its secret credentials. The node compromise consequently allows an adversary to be capable of creating clones or replicas of the compromised nodes and then surreptitiously deploying them at strategic positions of the network.

An important distinctive behavior of clones or replicas is that they act as legitimate nodes or authorized participants in the network. These clones have the cryptographic keying materials which allow them to seem like original legitimate sensor nodes. Since, they behave honestly and participate in the network operations like non-compromised sensor nodes so that the legitimate and honest nodes are not aware of that there is a clone node among them. Consequently, all the existing authentication techniques and secure network communication protocols [3–5] would easily allow these replicas to create pair wise shared keys with other nodes and the base station, also enabling them to encrypt/decrypt all their communications. If these clones are not detected efficiently, swiftly and promptly, an adversary can easily take control of the network by exploiting these clones. Moreover, he/she can cripple many applications of the WSN as it is very easy for him/her to compromise and replicate a typical sensor node by using a few readily available tools [6] in a very short period of time. Also, once an adversary captures and compromises a single sensor node, it becomes very cheap to make clones and thus the main cost of attack is maintained. An adversary can also leverage these clones for launching many insider attacks and malicious activities. For example, he/she can create a black hole, initiate a wormhole attack when several clones team up together, launch selective forwarding attack and DoS attack, inject false data, monitor and overhear significant portion of traffic, denigrate and offend other nodes and even terminate legitimate nodes [7–8].

The most simple but unassertive solution to deal with these clone node attacks is to equip the sensor nodes with a tamper resistant hardware but this solution is inappropriate because of two main reasons; first, it is uneconomical and very expensive to shield each of the sensor nodes in the network with a tamper proof hardware, and second, it may still be possible to bypass tamper resistance for an expert attacker. Therefore, there is a need to develop software based countermeasures for the detection of clone nodes. In the literature two types of software based solutions have been proposed for the detection of clone attack in static WSNs namely *Centralized* and *Distributed*.

**Fig 1:**
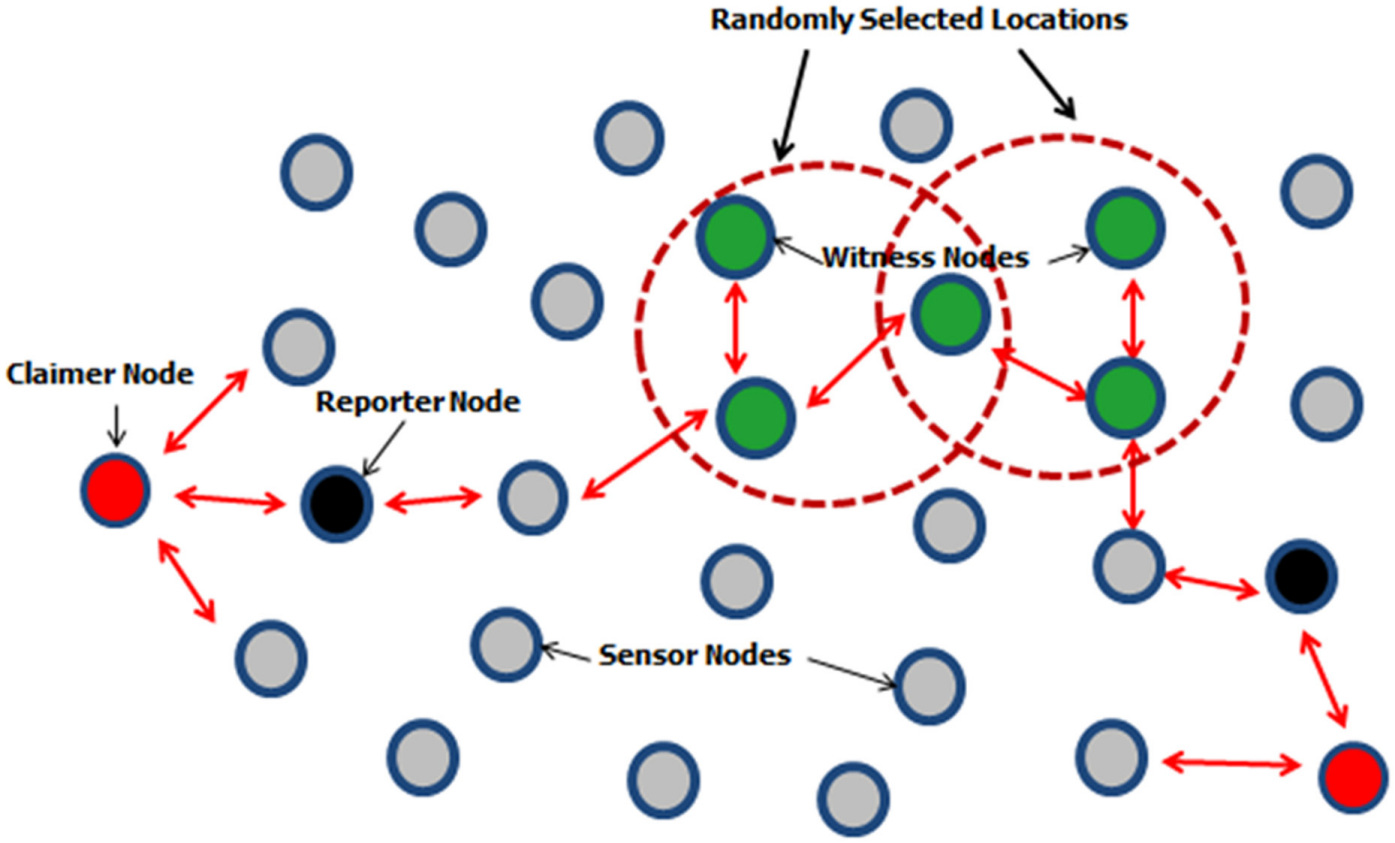
Working of Witness node based techniques following the claimer-reporter-witness based framework.

In *Centralized* solutions, the detection process is based on a base station [9–15] or assisted central authority (i.e. base station, cluster head etc.) [16–17]. Centralized solutions have achieved high clone detection rates but they all suffer from several drawbacks like single point of failure and high communication costs. Also the local protocols become inept to detect clone nodes which are distributed in different areas of the network. This diverted the attention of researchers towards distributed solutions. In *Distributed* solutions [18–23], the detection process is carried out by all the sensors nodes in the network without the involvement of any central authority. So far, the most promising distributed techniques to detect clone attacks are witness node based techniques [24–32] which have used claimer-reporter-witness framework to detect clones/replicas. In these techniques the claimer node locally broadcasts its location claim to its neighbors and each neighbor serves as a reporter node whose responsibility is to map the claimer *id* to one or more witness nodes. These witnesses detect clones upon receiving conflicting location claims. The witness node based techniques follow the claimer reporter witness framework which is shown in Fig 1.

The existing witness node based techniques can fundamentally try to detect clones but they are not completely satisfactory and suffer from significant problems like either the witness node selection is deterministic or the distribution of witness nodes over the network is non-uniform (for each iteration of the protocol). As a result, an adversary can attempt to attack and clones become undetected making detection technique useless. In case of deterministic witness node selection like Deterministic Multicast (DM) [24] an adversary can launch *smart attack* and thus able to guess about the witness nodes, easily compromising them. Moreover, the non-uniform distribution (like in Randomized Multicast (RM) [24]) of the witness nodes over the network can perish the sensor nodes as their energy is depleted soon by repeated selection of these nodes as witness nodes. The *masked replication attack* can also be launched against witness node based strategies which the entire existing witness node based techniques yet cannot detect. However, some neighbor cooperation techniques can be merged with witness nodes based techniques to help in detecting clones [33–38]. In witness node based techniques *witness nodes* are the fundamental elements as they have the power and responsibility of making important decisions, so, it is very essential and critical to ensure the security of witness nodes in the witness node based techniques. Ideally, clones can be detected efficiently by uniformly distributing the witness nodes over the network and selecting them in such a smart manner that an adversary won’t be able to judge that which will be the witness node. Thus, there is a need to develop such a protocol that fulfills these conditions, while keeping the communication and memory costs nominal also.

Amongst all the research efforts contributed so far for detecting clones in WSNs, Random Walk (RAWL) [28] is well thought to be the most promising witness node based solution. RAWL introduces the concept of random walk and witness nodes are randomly selected by initiating several random walks throughout the network, solving the drawbacks of other witness node based strategies [24] [16–17]. Although RAWL has achieved high security of witness nodes but it suffers from some noteworthy limitations. *First*, in accomplishing higher detection probability of clone nodes and stronger security properties, RAWL trades increased communication and memory overheads. *Second*, for ensuring to achieve the intersecting witness nodes, RAWL requires to initiate more random walks with long walk steps. *Third*, RAWL needs more reporters in order to initiate more random walks which will forward the location claim to randomly selected witness nodes all of which in turn initiate their respective random walks, each passing node also becoming the witness node. This motivated us to present an approach for detecting clones in WSNs in a more effective way to increase the security of witness nodes while keeping the communication and memory costs moderate.

In this paper we present a novel distributed solution called Random Walk with Network Division (RWND) which mingles the division of the network into areas with a random walk. It is based on claimer-reporter-witness framework and works in two phases. In the first phase called network configuration phase, the entire network is divided into hierarchical levels by using heuristic based algorithm and then one or more levels formulate a specific area. Each node in the network belongs to a certain level and area. In the second phase which is called clone detection phase, the claimer node sends a signed location claim to its one hop neighbors. One or more neighbors (reporter nodes) forward the claim to randomly selected nodes in any combination of randomly selected areas (we will describe the details of area selection in section IV) with some probability. The reporter(s) of a claimer node will select a single node randomly in each area which will further select r nodes randomly, that will finally initiate the random walks and the passing nodes at each random walk step will become the witness nodes. These witness nodes will finally store the location claim. If there are clones in the network they will forward the location claim in similar manner and if any witness node receives different location claims for the same node, a conflict is detected and finally a clone node will be revoked. We also analyze the proposed network division, area selection mechanism and the required number of walk steps for the detection of clones. We also perform security analysis of the proposed scheme to verify its resiliency against smart attacker. We evaluate the efficiency and performance of proposed scheme by performing extensive simulations under different settings and examining the detection probabilities and the communications and memory costs, comparing them with the existing witness node based RAWL. The simulation results show that our proposed protocol RWND outperforms the existing solution RAWL by reducing the communication and memory costs with higher detection probability.

The paper is organized as follows: In Section II we discuss some recent existing approaches which are closely related to our work. Section III presents some vital requirements which are essential for distributed witness based techniques. In Section IV, we describe the network and adversary models used for our proposed scheme. After analyzing some important drawbacks of existing approaches we present our proposed protocol in Section V. In section VI we theoretically analyze of proposed network division and areas selection mechanism and required number of walk steps for clone detection. Section VII presents the simulation results and finally in Section VIII we conclude the paper.

## Related Work

One of the first attempts for clone detection was a centralized one proposed in [39] which was the most straightforward and naïve solution relying on a base station or assisted central authority. Some of the other centralized solutions proposed so far can be found in [9] [10] [12] [15] and [16–17]

The first naïve distributed solution for detecting clones is Node-to-Network broadcasting (N2NB) [24] in which a message containing the location information is flooded in the network by all the nodes and then the received location information is compared with their neighbors. A clone is detected upon receiving a conflicting claim and is isolated from the network after the revocation process. Some distributed approaches proposed to detect clone attacks have used claimer-reporter-witness framework and are also called witness node based techniques [24–32], that are most promising strategies so far. However all of them still suffer from some limitations.

B.Parno et al. [24] were the first to propose two probabilistic algorithms Randomized Multicast (RM) and Line-Selected Multicast (LSM) for the full fledge detection of clones/replicas in wireless sensor networks which follow the claimer reporter witness approach. In RM, when a claimer node announces its location by locally broadcasting the signed location claim to its neighbors, each of its neighbor nodes (who is aware of its own position) become a reporter with probability *p* after verifying the plausibility of the location. Each reporter then selects $O(\sqrt{n})$ random destinations in the network and forwards the authenticated location claim to the nodes close to those random locations that are called witness nodes. If there is a replica in the network and the reporters of that replicated node also select $\left(\sqrt{n}\right)$ random destinations then by exploiting the birthday paradox at least one common witness will receive two conflicting location claims with high probability. This witness node can immediately publicize the network with the evidence of incoherent location claims to discredit and revoke the clone/replica node. RM implies high communication cost as each neighbor has to send $O(\sqrt{n})\;$ messages to achieve common witnesses. In LSM, when a claimer node announces its location, every neighbor becomes a reporter with probability p after locally checking the signature of the claim and then forwards it to randomly selected destinations. During the propagation of the location claim from a reporter node to a witness node it must pass through several intermediate nodes on the forwarding route which also store the location claim randomly drawing a line across the network and thus serve as additional witnesses. In case of a clone node, when a conflicting location claim by a clone node crosses the forwarded path for the legitimate node, the intermediate node at the intersection of the two paths will detect the conflict, further excluding the clone node. LSM was proposed to introduce an improvement in terms of detection probability and the network-wide communication cost reduced from $\left(n^{2}\right)\;to\;O(n\sqrt{n})$. But LSM suffers from uneven distribution of witnesses nodes as majority of witness nodes are selected from the center of the network, the energy of these nodes is depleted soon thus they become the point of interest for the adversary.

Zhu et al. [25][26] have proposed two distributed protocols called Single Deterministic Cell (SDC) and Parallel Multiple Probabilistic Cells (P-MPC) with the purpose of increasing the detection probability attained by LSM for detecting node replication attacks. The notion of both protocols is to form a geographic grid by logically dividing the whole network into cells, considering all the nodes within a cell to be the possible witnesses. In SDC, each node ID is uniquely mapped to a single cell and for broadcasting the location claim within the cell the location claim is first forwarded from the claimer node to its reporters which forward the location claim with a probability to a unique cell by executing a geographic hash function [40] with the input of node’s ID. Once any node in the destination cell receives the location claim, it floods the location claim in the entire cell; each node is probabilistically chosen to be a witness by saving the claim. In P-MPC the location claim is mapped and forwarded to multiple deterministic cells with various probabilities. The rest of the procedure is similar to SDC. Convincingly the most critical issue is that both of these techniques depend upon the careful selection of a cell size (*s*) as when the cell size (s) is too large, they incur high communication cost and when *s* is too small an adversary can physically capture and easily compromise all the nodes in the cell. Another important issue with SDC is that for lessening the broadcast overhead, SDC requires to execute the flooding of location claim only when first copy of a node’s location claim reaches at the cell, ignoring the subsequent copies. In doing so, if the node that first receives the location claim does not happen to become a witness node it will be unable to distinguish between the claims of the legitimate and the clone node. RWND differs from SDC and P-MPC in that, in SDC and P-MPC the cell selection is deterministic and mapped through node’s ID. Also the location claim is broadcasted in the whole cell for witness selection whereas in RWND we divide the network into areas and the selection of areas for initiating random walks is fully random. The witnesses are selected using random walk within each selected area.

**Fig 2:**
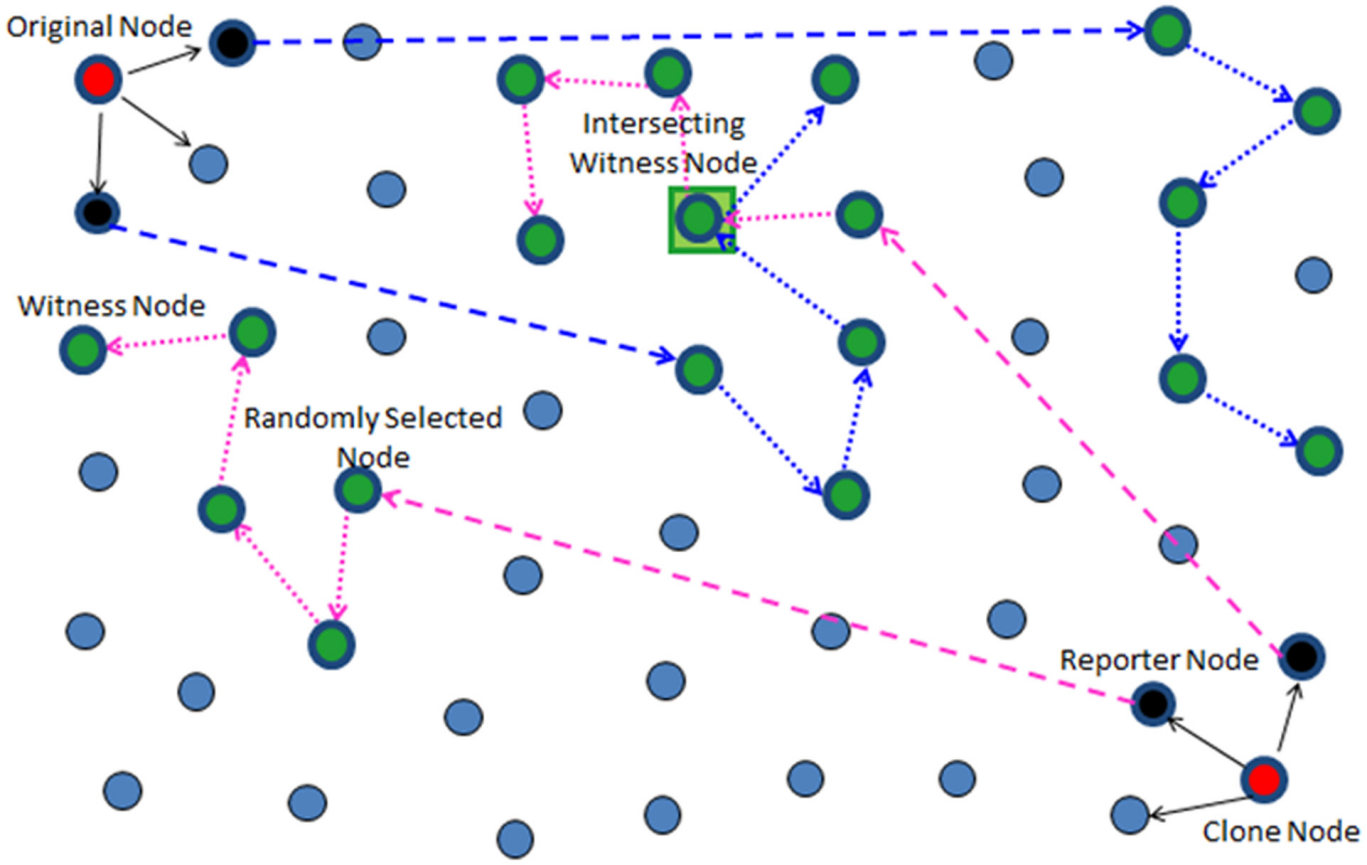
Working principle of RAWL.

Y.Zeng et al [28] have proposed two protocols Random Walk (RAWL) and Table-assisted Random Walk (TRAWL) for the detection of clone attack in wireless sensor networks. The Random Walk (RAWL) starts several random walks randomly in the network for each node a, and then selects the passed nodes as the witness nodes of node a. RAWL works in four steps in each execution. In the first step each node broadcasts a signed location claim. In the second step each of the node’s neighbors probabilistically forwards the claim to some randomly selected nodes. In the third step each randomly selected node sends a message containing the claim to start a random walk in the network, and the passed nodes are selected as witness nodes and will store the claim. In the fourth step if any witness receives different location claims for a same node ID, it can use these claims to revoke the replicated node. Their second protocol, TRAWL is based on RAWL and adds a trace table at each node to reduce memory cost. The RAWL needs more random walks and random walk steps for achieving high detection probability that leads to higher communication and memory cost which is more than twice the communication overhead of LSM. The authors reduce the memory cost by proposing TRAWL but the communication cost still exists. Fig 2 shows the working of the RAWL protocol.

Both RAWL and TRAWL follow a similar strategy (i.e. using random walk) for selecting witness nodes, differing in that TRAWL reduces the memory costs by employing trace table. In this paper we choose RAWL for comparison with RWND, because first it is the most promising solution so far, and second RAWL has used random walk for the selection of witness nodes. In RWND we also employ simple random walk but in an entirely different manner for selecting witnesses i.e. by combining network division with a novel witness selection method (for details see Section IV and V).

In this paper we review the contribution in [29] and after further investigating the RWND protocol by theoretically analyzing the area division and area selection mechanisms, a new witness node selection mechanism is introduced. The analysis and further simulations prove that RWND outperforms the previously proposed protocols in-terms of high clone detection probability and stronger security of witness nodes with moderate overheads. The other techniques for the detection node replication attack in static and mobile sensor network can be found in more details [41–43].

## Requirements for Distributed Witness Node Based Protocols

In witness node based strategies, critical witness (intersecting witness) nodes are an important element as these witnesses finally detect and revoke the clones in the network. The security of these witness nodes is indispensable as in deterministic protocols, it is relatively easy for an adversary to capture and replicate and then compromises the witness nodes because of the small number of witnesses. An adversary can succeed by keep on compromising these witness nodes during the lifetime of the network. To ensure the security of witness nodes, the selection of these witnesses should be non-deterministic and all the nodes in the network should have an equal probability of being witnesses. Consequently, it will be more difficult for an adversary to successfully launch clone attacks in non-deterministic protocols because the witnesses of a node are not known and are different in each execution of the protocol. Also the witness nodes should be uniformly distributed throughout the network and should not be selected repeatedly from any particular location of the network. These requirements should be fulfilled to guarantee the security of critical witness nodes which in turn increases the detection probability of clones.

As WSNs are resource constrained networks, both in terms of energy of nodes and their memory, it is thus very challenging to design protocols with little overhead. If the nodes start exhausting their batteries the whole network functionality is disrupted. Moreover, if only a small number of nodes are deliberated for high memory or storage purposes then these nodes can overflow which results in packet loss or packet dropping. This substantially affects the detection capabilities of the protocol. Hence it is very important to develop such protocols which incur average or moderate memory and communication overheads while utilizing the resources wisely and efficiently.

## Network and Adversary Models

In this section, we describe the assumed network and adversary models. The notations and symbols used in the paper are also presented.

### Network Model

We assume the sensor network in which a large number of low cost sensor nodes are uniformly distributed over a wide deployment area. All the nodes are assumed to know their own geographic locations by using some existing localization algorithms. Nodes are assumed to be stationary during the execution of clone detection protocol and also assigned a unique identity with a pair of identity based public and private keys. Same as previous works [16–17][24][28–29], we assume that adversaries cannot create sensors with new identities for replicas as any two nodes are also assumed to be protected by pair wise keys. New sensor nodes can be added into the network in order to replace the old ones and like [25–26] when a new node is added into the network, it needs to generate a location claim and broadcast the claim to its neighbors.

### Adversary Model

For an adversary model, we assume a simple but powerful adversary that is able to first capture and then compromise the sensor nodes. By using cryptographic information obtained from those compromised nodes he produces replicas/clones and then inserts them into the network. We also assume the existence of monitoring mechanisms or automated protocols like SWATT [44] which can draw human intervention and starts sweeping the network to remove compromised nodes if an adversary tries to compromise unlimited number of sensor nodes. Therefore we assume that an adversary may select only`limited number of nodes to capture and compromise.

### Notations

We list all the notations used in this paper in Table 1 for clarification.

**Table 1:** Notations & Symbols.

*N*_*n*_	Number of sensor nodes in the network
*N*_*a*_	Number of total areas in network
*N*_*sa*_	Number of selected areas by a reporter.
*N*_*c*_	Number of total combinations.
*I*_*a*_	Number of Intersecting area(s).
*S*_*a*_	Single selected area.
*d*	Average degree of each node / Number of neighbors of each node.
*P*_*fd*_	Probability of forwarding the location claim by a neighbor.
*P*_*d*_	Probability of Detecting Replica.
*r*	Number of random walks for each sensor node.
*t*	Number of walk steps by each random walk
*loc*_*a*_	Location information of a node (e.g. location (x, y) in 2D)
*ID*_*a*_	Identity of a node
*$K_{a}^{Pvt}$*	Private Key of node a
*$K_{a}^{Pub}$*	Public Key of node a
*$Sig{\left\{M\right\}}_{K_{a}^{Pvt}}^{}$*	Node ‘a’ signature on massage *M*
*H*(*M*)	Hash of massage *M*
*||*	Symbol for Concatenation

## Random Walk with Network Division (RWND)

In this section, a new distributed protocol called Random Walk with Network Division (RWND) for the detection of clones (node replication attack) is proposed, which is based on claimer-reporter-witness framework and combines the idea of simple random walk with network division.

### Protocol Description

RWND is an amalgamation of random walk and the network division. At a high level, RWND works in two phases, the *network configuration phase* and the *clone detection phase*. In the network configuration phase, the entire network is divided into hierarchical levels and then one or more levels formulate a specific area. Each node in the network belongs to a certain level and area. In the clone detection phase, the clone is detected by following a claimer-reporter-witness framework and employing a random walk within an area. In each execution, each node broadcasts a signed location claim to its neighboring nodes (called reporter node), each of which probabilistically forward the claim to some randomly selected nodes from a combination of randomly selected areas. The reporter(s) of a claimer node will select a single node randomly in each area which will further select *r* nodes randomly, that will finally initiate the random walks and the passing nodes at each random walk step will become the witness nodes. The selection of witness nodes in each area is random and different in each iteration of the protocol. The network division into moderate sized areas confers a large improvement over RAWL in terms of communication and memory costs as the required number of random walks and random walk steps are reduced. Moreover, the higher security of witness nodes is achieved by employing and initiating parallel (multiple) random walks within a combination of randomly selected areas. The formal description of each phase of our protocol is elaborated below.

#### Network configuration phase

In our proposed protocol, each node in the network belongs to a certain level with respect to a particular sink (because multiple sinks may exist in a network, without losing generality, only one sink or reference node is used) or any reference node. Here, the level represents the distance (in terms of hop count) to the assigned sink and each area consists of a different number of levels, depending on the sink configuration. We assume that the number of levels in each area is static and is configured during the network configuration phase depending upon the size of network. The idea of dividing the network into levels and areas is inspired by [45].

**Fig 3:**
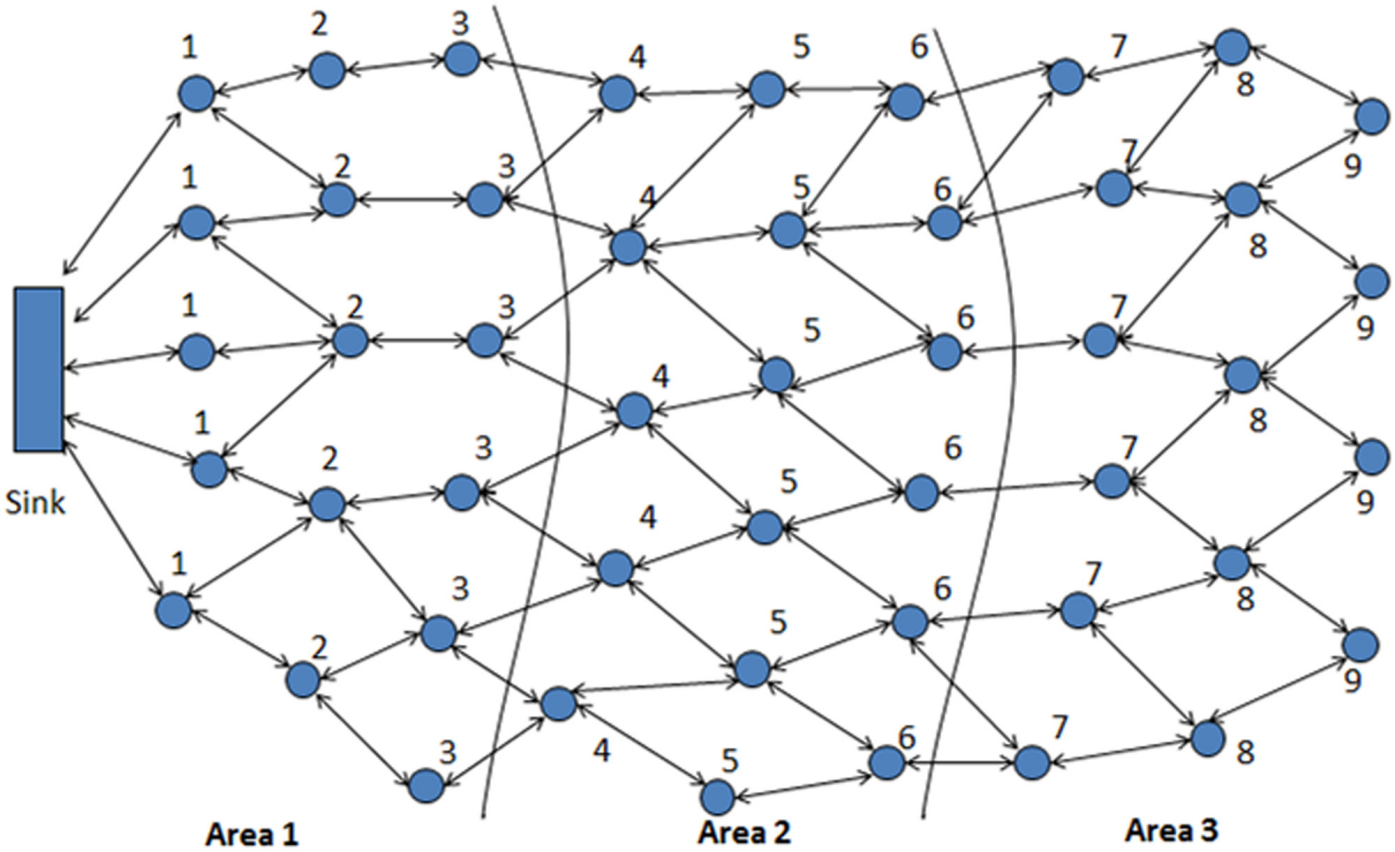
Level & Area Assignment during Network Configuration Phase.

The tagging process is the process of dividing the whole network into different levels and then the assignment of these levels to all the nodes. It is always initiated by the sink or reference node. A message is sent by the sink or reference node to its one hop neighbors and it contains sink number/ reference node *id*, and level/area. After receiving the message, each node broadcasts a message to report that it belongs to level one. All other nodes that listen to this message and do not have this information yet, will increase the value of the received level by one, assign themselves to this level and check their area before they broadcast this new level. This procedure continues until all nodes belong to a level and are assigned to an area. Once a node has assigned itself to a level and an area, it ignores all future broadcasts with level and area information. Fig 3 shows the above mechanism of level and area assignment to nodes during the network configuration phase.

#### Clone detection phase

This phase works in four stages by following the claimer reporter witness framework; Claim forwarding, Area selection, Witness node selection, Clone detection and revocation.

*1*. *Claim Forwarding*. At the first stage when the clone detection process starts, each node forwards a signed location claim to its neighbors and becomes a *Claimer Node*. The format of the location claim is: $\langle ID_{a},loc_{a},Sig{\left\{H(ID_{a}||loc_{a})\right\}}_{K_{a}^{Pvt}}\rangle$; where || denotes the concatenation operation and *loc*_*a*_ is the location information of node *a*.

**Fig 4:**
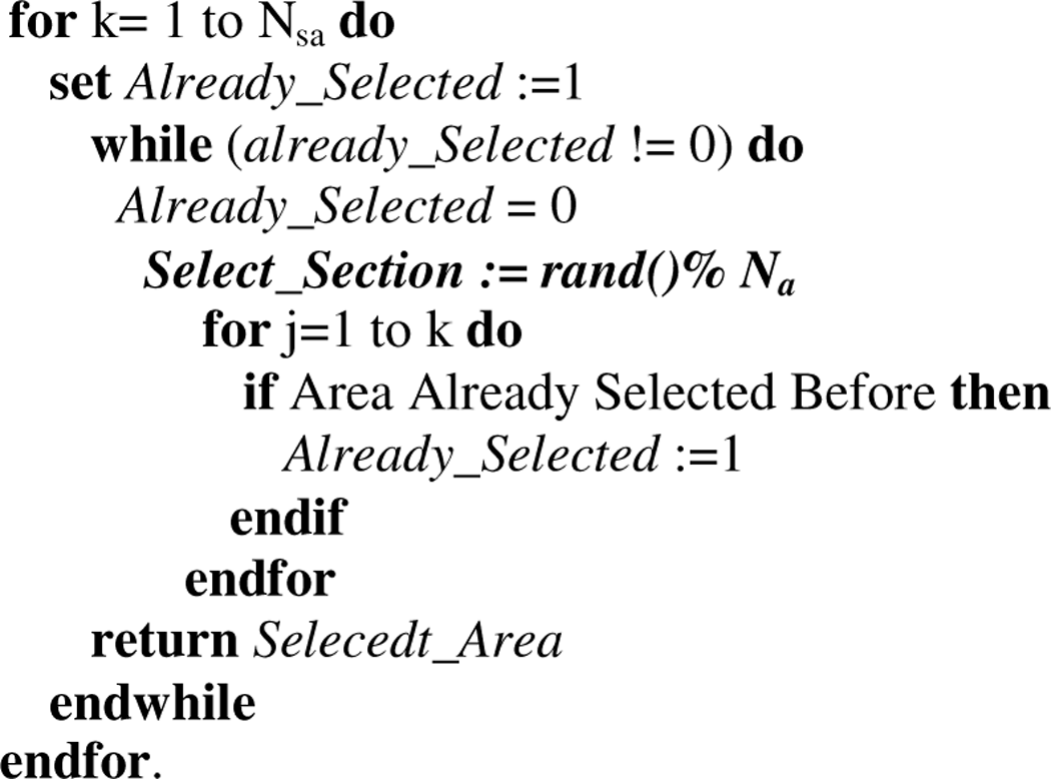
The pseudo code for the area selection mechanism.

*2*. *Area Selection*. After hearing the claim, each neighboring node first verifies the signature and the plausibility of the location of a claimer node (e.g. the distance between claimer and the neighboring node should be within the transmission range). The neighboring nodes will become the *Reporter Nodes* of that claimer node with some probability. The reporter node(s) performs two steps. In the *first* step which is called *Area Selection*, reporter(s) from any particular area forwards the location claim of a *claimer node* to some randomly selected areas using an area selection mechanism. This mechanism first defines the number of areas that are selected by the reporter node(s) from the total number of areas of the network for the purpose of forwarding the location (calculated according to Eqs 1 and 2 in analysis section). For any number of areas there are some possible combinations (calculated in detail in Analysis Section) which are unordered and without replacement. After selecting the number of areas, the reporter node(s) then randomly select any one combination of areas for forwarding the location claim. By following this procedure the reporter(s) from any area of the network can select any combination of areas which results in at-least one intersecting area (common area). Fig 4 shows the pseudo code for area selection.

**Fig 5:**
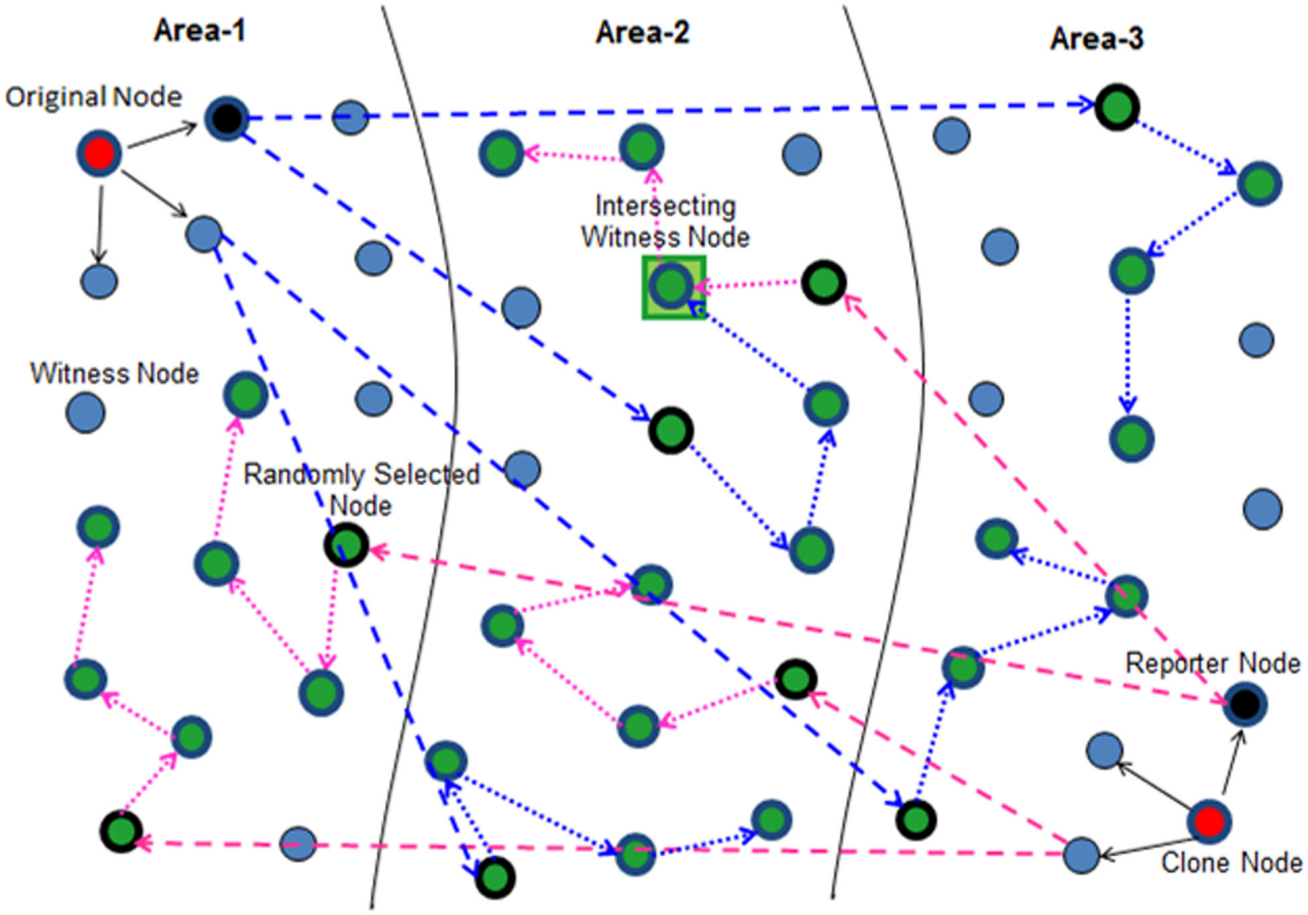
Working and witness node selection of RAND (our previous work [29]).

*3*. *Witness Node Selection*. In RAWL, the witness nodes are selected randomly by the reporter node(s) which further initiate *r* random walks in the whole network followed by *t* random walk steps and then each passing node also become the witness nodes. In our previous work [29], whose working is also shown in Fig 5, the reporter node(s) randomly selects *g* (where *g = r*) geographic locations by employing geographic routing protocols (GPRS [46]) with probability in order to forward the claim to the *g* locations (as according to [17], [28] choosing a random location is far better and more secure than choosing Node ID) in each randomly selected area. In doing so, although the required number of random walk steps was greatly reduced which in turn reduces the overall communication cost to some extent as compared to RAWL due the division of the network into small areas but the communication cost of reporter to randomly selected nodes had increased as the reporter has to randomly select *r* nodes in each selected area (selected by the reporter according to Eq 1) for initiating the *r* random walks.

**Fig 6:**
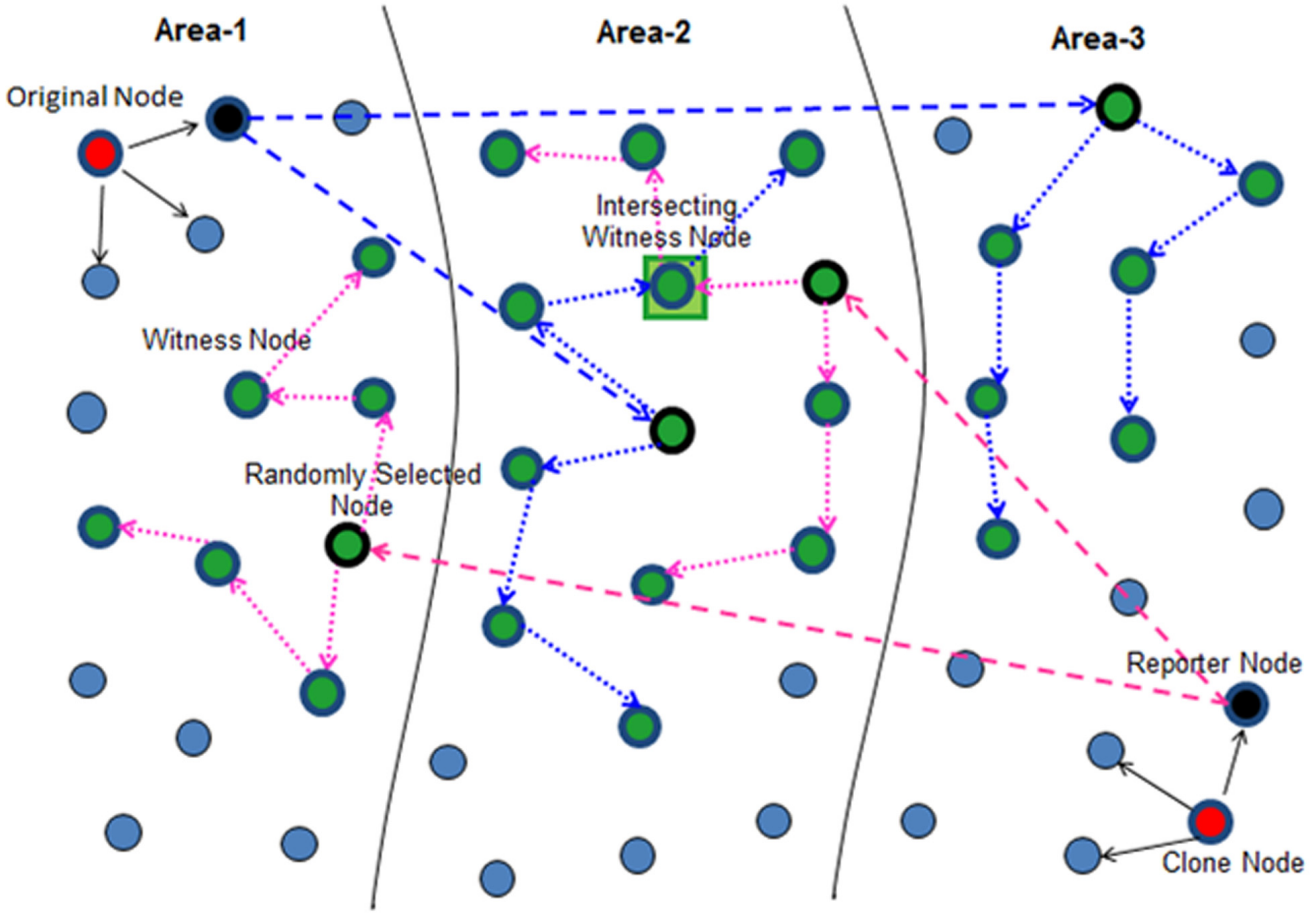
Working principle of RWND (With Improved Witness Detection Mechanism).

The *second* step that each reporter performs, defines the *Witness Node Selection* mechanism which is enhanced in this paper for achieving greater security of witness nodes as well as to reduce the communication cost. In this new mechanism, the reporter(s) of a claimer node will select a single node randomly in each selected area each of which receive the location claim and after verifying the signature each of them will become the witness nodes after storing the location claim and will finally initiate the *r* random walks of *t* walk steps, the passing nodes at each random walk step also becoming the witness nodes. Fig 6 shows the improved witness node selection mechanism.

This new witness node selection mechanism minimizes the cost of reporter to randomly selected node in each area finally resulting in reduced communication cost of our scheme which is main motive of this mechanism. The simulation results verify that this proposed mechanism of witness node selection has reduced the communication costs for achieving higher detection probability as compared to our previous work and as well as RWAL.

*4*. *Clone detection and revocation*. When a witness node finds a conflict (two different location claims with same node ID) it will revoke the clones by broadcasting the two conflicting claims as an evidence. Every node will terminate the links with clones after they independently verify the signatures.

#### Security analysis of RWND

RWND satisfies all the above ideal requirements of witness node based techniques (as described in “requirements for distributed witness node based protocols” section), as we divide the network into number of areas and random walks are initiated in randomly selected areas (any reporter can select any area with equal probability (i.e. 1/*N*_*a*_) providing an additional layer of security. In these randomly selected areas, a random node is selected which will further select some random nodes in each area to initiate (*r*) random walks, each passing node becoming the witness nodes. In result a smart adversary is unable to find out the critical witness nodes. The confrontation of RWND can be instinctively explained by that it offers an additional layer of security of witness nodes through an area selection mechanism and then the responsibility of witness node selection is distributed to every passed node of random walks.

In witness node based techniques (claimer-reporter-witness based techniques), if an adversary compromises all the witness nodes of a captured node, he can easily and safely deploy any number of clones or replicas in the network. Also an adversary may try to protect his replicas by launching a smart attack in which he first finds out the replica detecting witness nodes and then only compromises these witness nodes. In order to avoid these attacks, the security of witness nodes must be ensured and the witness nodes should be selected non-deterministically and randomly in a way that each node (in each round or iteration) in the network has an equal probability of being a witness node without the involvement of a central authority [16–17]. In LSM, nodes in the center of the network are mostly selected as witness nodes (called crowded center problem) and thus an adversary can succeed to capture and compromise the witness nodes. In RWND the witness nodes are selected non-deterministically and fully randomly by dividing the network into areas and selecting the witness nodes for each node randomly from different areas using a random walk.

In case of a smart adversary that aims to discover and compromise all the critical witness nodes, there may be a possibility that he learns from the reporter node about its randomly selected node (starter node) in each area from where the random walk starts. In order to learn about the next witness node, he has to scan and compromise all the ***d*** neighbors of the current witness node and for ***t*** random walk steps an adversary has to keep on compromising witness nodes for ***t*** times. Thus the total number of nodes needed to be compromised for a particular node is $O\left(\sqrt{\frac{N_{n}}{N_{a}}}\;\log\sqrt{\frac{N_{n}}{N_{a}}}\right),\;$ which is beyond the adversary’s ability (also we have assumed that an adversary can compromise only a limited number of nodes).

## Analysis

In this section, we theoretically analyzed the division of the network into different areas, the proposed area selection mechanism, and the required number of walk steps for the detection of clones.

### Analysis of Network Division

According to our network configuration phase, we divide the whole network into small areas and every node belongs to a particular area. Suppose we divide the network into (N_a_) number of areas (e.g. A_1_, A_2_, A_3_, A_4_…n). We can divide the network into any number of areas but the area division is dependent on many factors i.e. the security of witness nodes, size of areas, overall communication cost and size of the network. In the network configuration phase, we assume the minimum number of areas as N_a_ = 3. This is because if we divide the network into 2 areas then according to the proposed area selection mechanism each reporter will select both of the areas which make it easy for an attacker to know about the critical witness nodes. In result he can effortlessly compromise the whole area to shelter his clones or to evade detection. In case of N_a_ ≥ 3 areas, each reporter selects areas in a fully random fashion as they have more than one ways for selecting different combinations of areas, thus, it is very hard for an attacker to guess about which areas reporter will select. Consequently, greater security of witness nodes can be achieved by dividing the network into minimum of N_a_ ≥ 3 areas.

The size of each area is significant as if the network is divided into areas of small magnitude then it can be very easy for an adversary to launch clone attack. Thus it is essential to investigate the possible smallest and largest size of an area in which the network is divided so that the security of witness node remains high. As the size of each area depends upon the total number of nodes in the network (size of the network) so the optimal approximate size of each area can be calculated by using the formula $\left(S_{a}\;=\;\frac{N_{n}}{N_{a}}\;\approx \;\sqrt{N_{n}}\ln{(N}_{n})\right)$. The maximum number of areas in the networks can be calculated by using the formula $(N_{a}\approx \frac{N_{n}}{\sqrt{N_{n}}\ln{(N}_{n})})\;.\;$ Both of these depend upon the total size of the network. Fig 3 shows the network division into three areas.

### Analysis of Area Selection

During the detection process when a claimer node sends its location claim to its neighbors, the neighbors with some probability will forward the location claim to randomly selected nodes located at different areas in which the network is divided into. Each reporter node performs two steps. In the *first step* which is called area selection, every reporter randomly selects area(s) through our area selection mechanism which defines how many numbers of areas are selected by the reporter nodes from the total number of areas of the network for the purpose of forwarding the location claim to randomly selected nodes in those randomly selected areas. The area selection mechanism is dependent upon the number of areas into which the whole network is divided. If the whole network is divided into odd number of areas (3, 5, 7 and so on) the reporters will randomly select areas according to Eq 1 and if the whole network is divided into even number of areas (4, 8 and so on) the reporters will randomly select areas according to Eq 2. Eq 3 shows the general equation for area selection.

$$\text{Areas to Select }\left({\text{N}}_{\text{sa}}\right)=\left(\frac{{\text{N}}_{\text{a}\left(\text{odd}\right)}+1}{2}\right)$$ (1)

*where N*_*a*(*odd*)_ = {2*n* + 1,∀*n* ∈ *N* /*N*_*a*(*odd*)_ ≥ 3}

$$\text{Areas to Select }\left({\text{N}}_{\text{sa}}\right)=\left(\frac{{\text{N}}_{\text{a}\left(\text{even}\right)}}{2}+1\right)$$ (2)

*where N*_*a*(*even*)_ = {2*n*,∀*n* ∈ *N* /*N*_*a*(*even*)_ ≥ 4}

$$\text{Areas to Select }\left({\text{N}}_{\text{sa}}\right)=\left\lfloor \frac{{\text{N}}_{\text{a}}+1}{2}\right\rfloor$$ (3)

Where N_a_ is the total number of areas into which the whole network is divided. For any number of areas there are some possible combinations which are unordered and without replacement. The number of ways in which each reporter can select a number of different areas (*N*_*sa*_) out of the total (*N*_*a*_) areas are Nc = NaNsa. The total number of possible combinations can be calculated according to Eq 4.

$$\begin{array}{l} N_{c}=\left(\begin{array}{c} N_{a}\\ N_{sa} \end{array}\right)=\;\frac{N_{a}\left(N_{a}-1\right)\left(N_{a}-2\right)\ldots \left(N_{a}-N_{sa}-1\right)}{N_{sa}\left(N_{sa}-1\right)\left(N_{sa}-2\right)\ldots 1}\\ N_{c}=C_{N_{sa}}^{N_{a}}=\frac{N_{a}!}{N_{sa}!\left(N_{a}-N_{sa}\right)!} \end{array}$$ (4)

In witness node based mechanisms, the core component is the selection of witness nodes and the whole procedure of clone detection is based upon how these witnesses are selected. The criterion of selecting witness nodes is not only responsible for witness distribution and detection probability but also protects the critical witness nodes from smarter adversaries. Conclusively the major aim of any witness node based mechanism is to secure the witness nodes in such a manner that it is impossible or even harder for any clever adversary to compromise the witness nodes. In order to achieve the above notion we propose to combine the network division into areas with random walk and provide an effective area selection mechanism for selecting witness nodes. The main objective of the above area selection mechanism is to provide added security against smarter adversaries (who can use brute force attack), so that they won’t be able (even harder) to guess about the areas in which the critical witness nodes have been selected. It also ensures the even witness distribution throughout the network.

After selecting the number of areas, the reporter nodes then randomly select any number of possible combinations of areas (using Eq 4) for forwarding the location claim. Each reporter will select single area out of the single combination of areas using Eq 5.

$$S_{a}=(Rand()\%\;N_{a})$$ (5)

By following the above mentioned procedure the reporters from any area of the network can select any combination of areas which results in at-least one intersecting area (common area). If the network is divided into odd number of areas, at least one intersecting area is achieved whereas if the network is divided into even number of areas then at least two intersecting areas will be achieved. So, the reporters of both the legitimate node and the clone node will get {1 ≤ (I_a_) ≤ N_sa_}intersecting area(s) for the selection of any combination of areas.

### Required Number of Walk Steps for Detection

In both RAWL and RWND, the number of random walk steps (*t*) is closely related to the detection probability of these protocols. In RAWL, the longer the walk steps, the higher the probability that the random walks for replicas intersect and thus the increase in random walks steps consequently raises the communication and memory costs, trading stronger security for increased communication and memory overheads. In RWND, the whole network is divided into almost equal areas in which parallel random walks are initiated with (*t*) random walk steps; passing nodes becoming the witnesses. The simulations results have shown that the less number of random walk steps are required by RWND for higher detection probability with moderate communication and memory overheads. As it is critical to determine the number of random walk steps (*t*) required for higher detection probability, so in this Section we would like to theoretically analyze the relation between the detection probability and the number of random walk steps (*t*).

For simplifying the analysis, we assume our network as a torus which is a grid graph (d-regular graph, d = 4) and is enclosed in both the north-south and east-west directions as in [28] [47–48]. The main objective is to find out the intersecting witness nodes (achieving at least one critical witness) which can be found by using two methods that exist in the literature. The first method is by determining the number of random walk steps that are required by any two random walks which are initiated by the reporters of a legitimate and its clone node in order to obtain the intersecting witness nodes. The second method is by calculating the number of passing nodes required by any two random walks to intersect at a common node.

In the first method walk steps are calculated by using hitting time. Hitting time is the walk steps required to hit a node. The hitting time of sensor node *a* is defined as the first time a random walk hits *a* when all random walks start from the stationary distribution (1/n, i.e. random walk starts from all nodes with equal probability) [48]. The average hitting time of random walk on tours is *O*(*N*_*n*_log*N*_*n*_) [48]. So the hitting time *H*_*a*_ for a node *a* can be approximated as follows [48][49]:

$$P\left(H_{a}>t\right)\approx \exp\left(\frac{-t}{cN_{n}log\left(N_{n}\right)}\right),$$ (6)

Where N_n_ is total number of sensor in the network and c ≈ 0.34 (constant) as N_n_ → ∞ valid for *N*_*n*_ ≥ 25 [49]. The hitting time for sensor node *a* where there are (*N*_*r*_ > 1) random walks in the network starting from a stationary distribution and is given by [47]:

$$P\left(H_{a}^{N_{r}}>t\right)\approx \exp\left(\frac{-t}{c\frac{N_{n}}{N_{r}}\log\left(N_{n}\right)}\right)$$ (7)

Details of the proof can be seen in [47]. Using the above hitting time, the authors of [28] have shown that $O(\sqrt{N_{n}}\log N_{n})$ steps are sufficient for the intersection of two random walks.

In the second method, if two random walks on the graph visit $O(\sqrt{N_{n}})$ distinct nodes, then according to birthday paradox [50], there is a high probability that these two random walks will pass through the same node [51][52].

As we know that single walk step refers to a sensor node, the above analysis concludes that the number of walks steps required for two random walks to intersect at the same node is between $O(\sqrt{N_{n}})$ and $O(\sqrt{N_{n}}\log N_{n})$. In case of RWND, we divide the whole network into number of areas (*N*_*a*_) and random walks are initiated in each selected area by the reporters of a claimer node and our area selection mechanism ensures that at-least one intersecting area will be selected by the reporters of a legitimate and its clone node if we divide the network into any number of areas (See Analysis Section for details). As the size of each area can be approximately $\left(S_{a}\approx \;\frac{N_{n}}{N_{a}}\right)$ that is much smaller than the whole size of a network so the less number of walk steps will be required for two random walks in a single area to intersect at a common node. So in RWND the required number of walk steps will be between $O(\sqrt{\frac{N_{n}}{N_{a}}})\;$ and $\;O(\sqrt{\frac{N_{n}}{N_{a}}}\;\log\sqrt{\frac{N_{n}}{N_{a}}})$, which is also verified by our simulations.

## Simulation Results

In this section we evaluate our protocol by simulations and performance is compared with existing protocol RAWL which has high communication and memory overheads owing to the longer random walks (or more random walk steps). We verify that RWND requires lesser number of walk steps with moderate communication and memory overheads than RAWL.

To facilitate a fair comparison, we used the same simulation methodology and simulation code that is used by Y. Zeng et al. [28]. We deployed 1024 nodes in 160 x 160 square grid areas. In our experiments we divided the network into 3, 4 and 5 areas and ran the simulations for 10,000 times for each random walk and walk steps randomly and exclusively. Parameters which were considered for simulations are shown in Table 2.

**Table 2:** Simulation parameters for Grid based Deployment Topology.

Parameters	Values
Deployment Area	160m x 160m
Number of Sensor Nodes	1024
Deployment/ topology type	Grid
Communication range	5m
Location Claim size	46 bytes
Average number of neighbors	4
Number of simulations runs	10000

### Required Number of Walk Steps for Clone Node Detection

In this subsection we simulate RWND and RAWL to confirm that certainly RWND needs much less number of random walk steps for achieving 95% detection probability. In both RWND and RAWL detection probability only depends upon intersecting witness nodes and usually a single intersecting witness node is enough for the successful detection of replica nodes. We calculated the probability of detection (*P*_*d*_) by using the following formula.

$$\left(P_{d}\right)\left(\%\right)=\;\frac{\left(Total\;\#\;of\;Successful\;Detection\right)}{\left(Total\;\#\;of\;Simulation\;Runs\right)}*100$$

**Fig 7:**
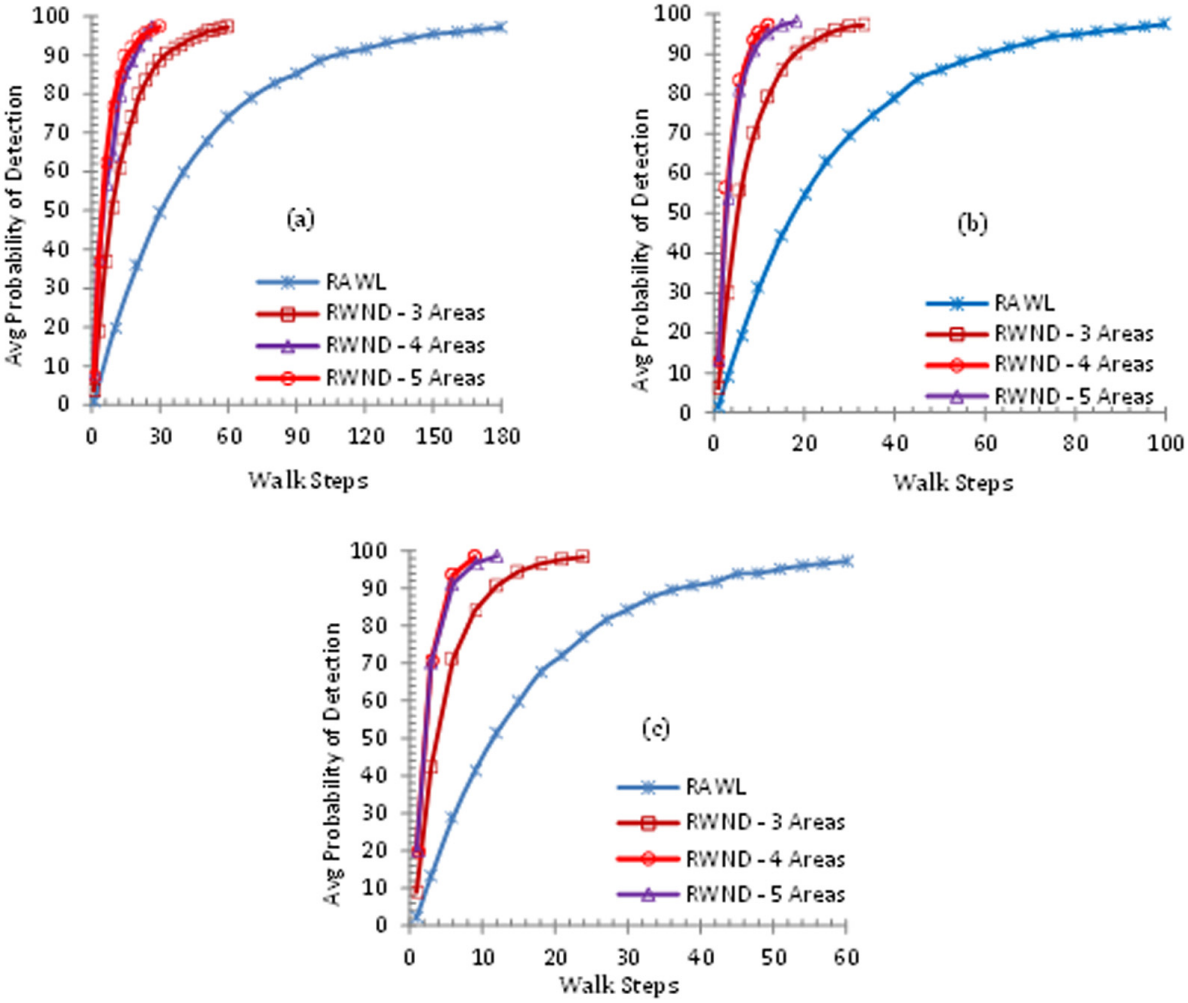
The Probability of Clone Detection, where the number of random walks (r) in 7(a) is 3, 7(b) is 4 and 7(c) is 5.

The number of random walk steps in RAWL and RWND is closely related to the probability of detection as Fig 7a, 7b and 7c show the increase in the walk steps results in higher detection probability for both RWND and RAWL. For RWND the network is divided into 3, 4 and 5 areas and Fig 7a, 7b and 7c demonstrate random walk steps needed to achieve more than 95% detection probability when random walk is r = 3, r = 4 and r = 5 respectively.

According to [51][52] 50% probability of intersection of two random walks is ensured if these two random walks pass through $\left(\sqrt{N_{n}}\right)$ distinct nodes by exploiting the birthday paradox algorithm. However for ensuring the detection probability up to 95%, there is a need to initiate multiple (more than two) random walks for each claimer node. In case of initiating multiple random walks the optimal length (random walk steps) of each random walk should be approximately $\left(\sqrt{N_{n}}\right)$ walk steps.

The increased number of walk steps will raise the detection probability of clone nodes but in addition it also increases the communication and memory costs. Hence, in RWND as we have divided the network into different areas so we need less number of random walk steps as compared to RAWL for getting intersecting witness nodes, resulting in achieving high detection probability.

### Communication & Memory Overhead

Communication in wireless sensor networks uses more energy than other operations [53]. The increase in random walks and random walk steps consequently raises the communication and memory costs. RAWL requires twice the communication overhead of LSM for achieving 95% detection probability thus they trade increased communication overhead for stronger security properties. In case of RAWL, two types of communication overheads are involved in the detection procedure, *first* is the communication cost incurred in forwarding the location claims from the reporters to the randomly selected nodes in the whole network. *Second* communication cost is incurred in initiating random walks by randomly selected nodes till the end of all random walk steps. RWND involves two types of communication costs. The first cost is incurred in selecting a single random node in each area by the reporters in each randomly selected areas and second cost is incurred in initiating *r* random walks by each randomly selected node (through its neighbors) in each randomly selected area.

The approximate average distance between any two randomly selected nodes in a randomly deployed network on a unit square is $\left(\frac{\sqrt{N_{n}}}{2}\right)$ [24]. For calculating memory overhead of both RWND and RAWL, we assume the size of location claim is about 46 bytes (similar to RAWL) i.e. for node ID (2 bytes), for location information (4 bytes), and for signature (40 bytes) e.g. ECDSA [54].

**Fig 8:**
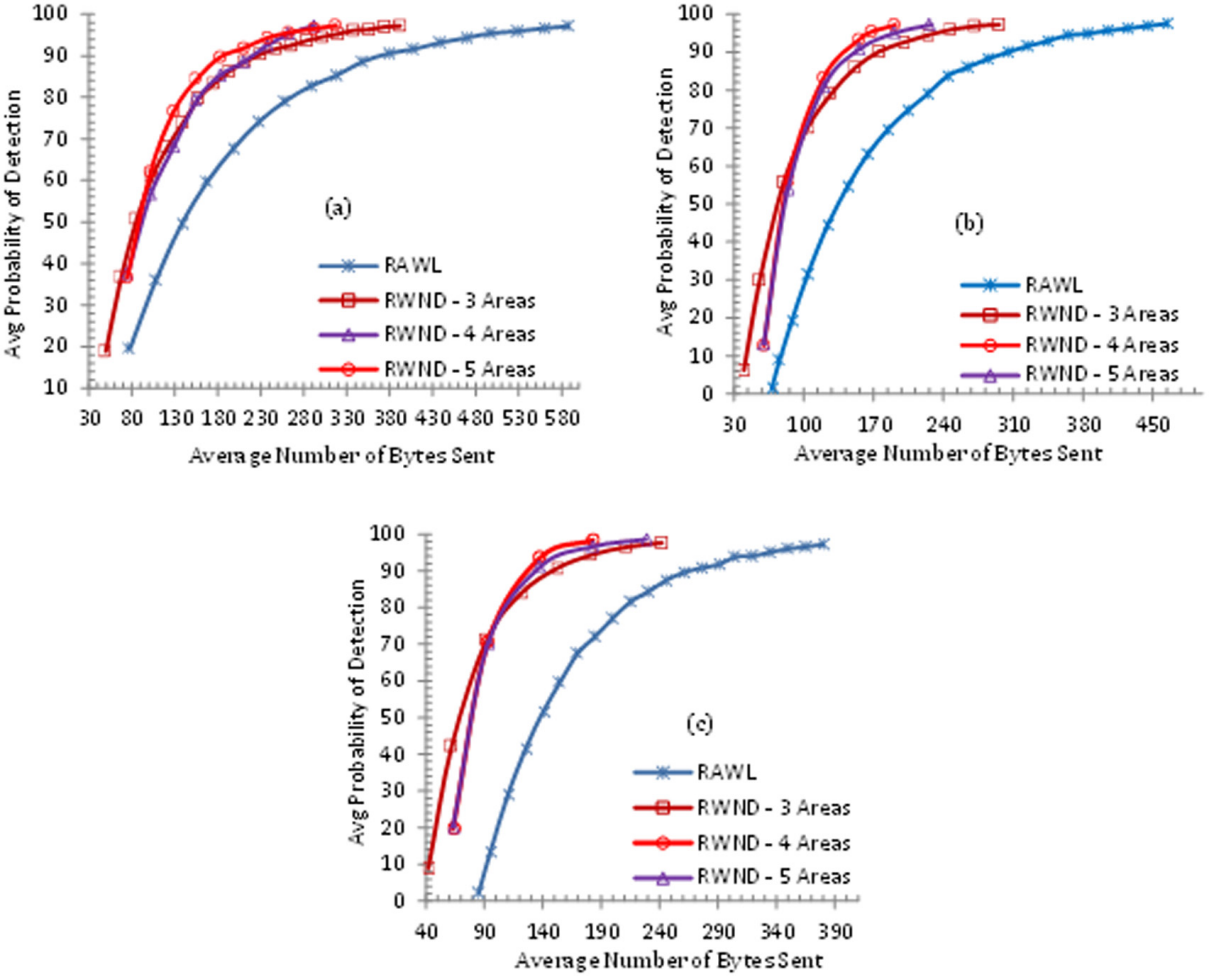
Comparison of Communication Cost of RAWL and RWND, where the number of random walks (r) in 8(a) is 3, 8(b) is 4 and 8(c) is 5.

**Fig 9:**
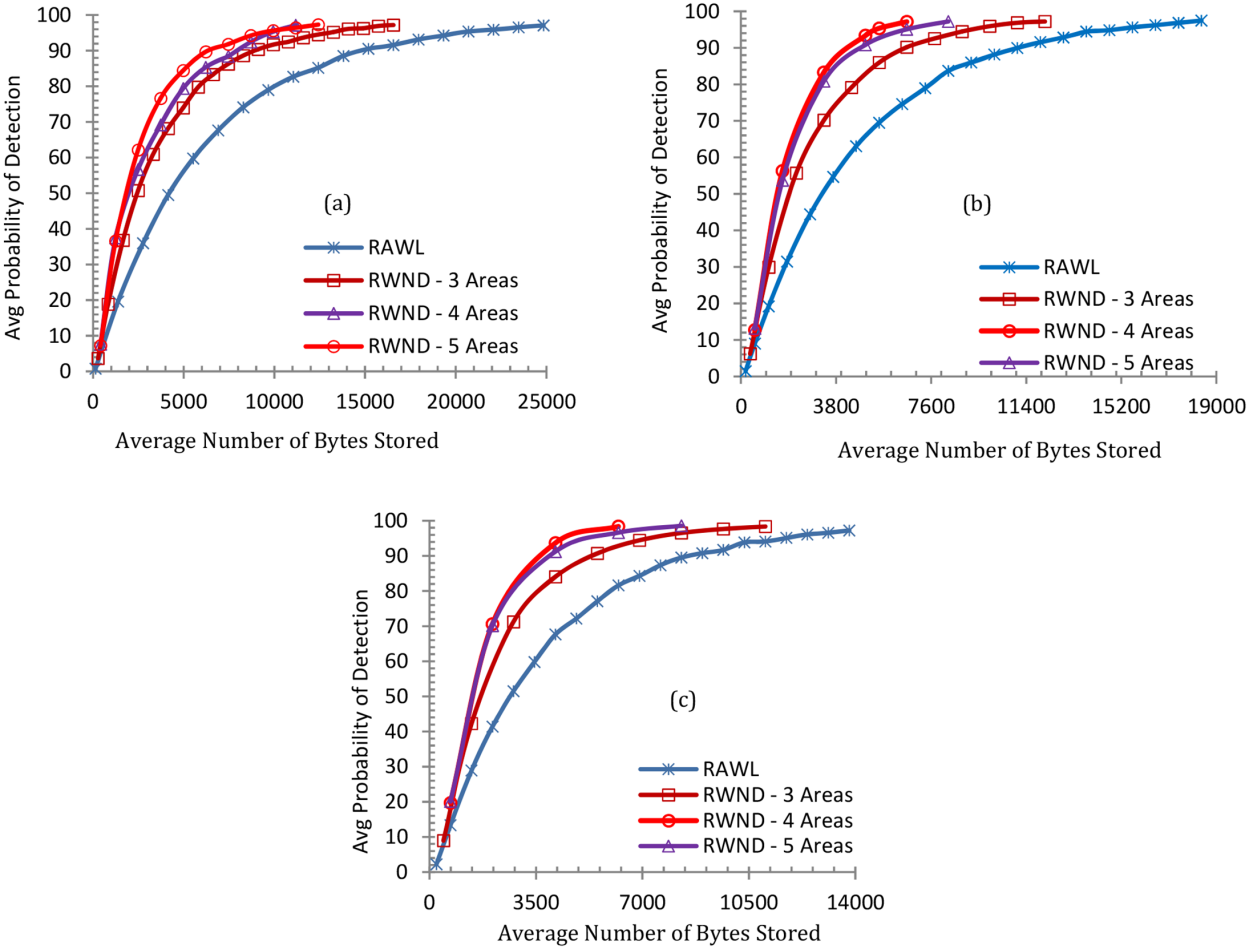
Comparison of Memory Cost of RAWL and RWND, where the number of random walks (r) in 9(a) is 3, 9(b) is 4 and 9(c) is 5.

Fig 8a, 8b and 8c demonstrate the communication overhead of both RWND and RAWL when *r* is 3, 4 and 5 respectively for each of the 3, 4 and 5 areas for the case of RWND. These clearly show that RWND consumes the lower communication overhead than RAWL for achieving 95% detection probability. Similarly Fig 9a, 9b and 9c show the memory overheads for both RWND and RAWL. These demonstrate that for achieving 95% detection probability, when *r* = 3, RWND requires 36%, 63% and 54% less memory than RAWL when the network is divided into 3, 4 and 5 areas respectively. RWND requires 41%, 64% and 57% less memory than RAWL when the network is divided into 3, 4 and 5 areas respectively with *r* = 4, whereas with *r* = 5, RWND requires 37%, 59% and 52% less memory as compared to RAWL when the network is divided into 3, 4 and 5 areas.

## Conclusion

In this paper we have presented a distributed witness node based scheme called RWND for the detection of clones in the static WSNs which is based on the claimer-reporter-witness framework. We have determined several important shortcomings of all the existing concomitant witness node based schemes. We have also pointed out and explained some significant weaknesses of the most recent and promising solution RAWL for the detection of clones attacks or node replication attacks. We have improved our previous work RWND by introducing a novel mechanism for the witness node selection as the witness selection is an important part of all the witness node based strategies on which the whole detection process of clones or replicas is reliant on. We have also provided a theoretical analysis of our proposed area selection mechanism, the security analysis of our whole scheme and the simulation results comparing our proposed scheme with RAWL. The extensive simulations confirm that our proposed protocol outperforms the RAWL protocol as the security of witness nodes is enhanced significantly with moderate communication, computation and memory overheads.

## Supporting Information

S1 Data (XLSX)

## References

[1] Akyildiz IF, Su W, Sankarasubramaniam Y, Cayirci E (2002). Wireless sensor networks: a survey. Computer networks, 38(4), 393–422.

[2] Khan WZ, Xiang Y, Aalsalem MY, Arshad Q (2013). Mobile phone sensing systems: A survey. Communications Surveys & Tutorials, IEEE, 15(1), 402–427.

[3] Zhu S, Setia S, Jajodia S, (2006). LEAP+: Efficient security mechanisms for large-scale distributed sensor networks. ACM Transactions on Sensor Networks (TOSN), 2(4), 500–528.

[4] Karlof, C, Sastry N, Wagner D, (2004). TinySec: a link layer security architecture for wireless sensor networks. In Proceedings of the 2nd international conference on Embedded networked sensor systems (pp. 162–175). ACM.

[5] Perrig A, Szewczyk R, Tygar J, Wen V, Culler D (2002) SPINS: security protocols for sensor networks. Wireless Networks, 2002; 8: 521–34.

[6] Hartung C, Balasalle J, Han R (2005). Node compromise in sensor networks: The need for secure systems. Department of Computer Science University of Colorado at Boulder.

[7] Karlof C, Wagner D (2003). Secure routing in wireless sensor networks: Attacks and countermeasures. Ad hoc networks, 1(2), 293–315.

[8] Wood A, Stankovic J (2002) Denial of Service in Sensor Networks. IEEE Computer. 2002 10; 3(10):54–62.

[9] Choi H, Zhu S, La Porta, TF (2007). SET: Detecting node clones in sensor networks. In Security and Privacy in Communications Networks and the Workshops, 2007. SecureComm 2007. Third International Conference on (pp. 341–350). IEEE.

[10] Brooks R, Govindaraju PY, Pirretti M, Vijaykrishnan N, Kandemir MT (2007). On the detection of clones in sensor networks using random key predistribution. Systems, Man, and Cybernetics, Part C: Applications and Reviews, IEEE Transactions on, 37(6), 1246–1258.

[11] Eschenauer L, Gligor, VD (2002). A key-management scheme for distributed sensor networks. In Proceedings of the 9th ACM conference on Computer and communications security (pp. 41–47). ACM.

[12] Xing K, Liu F, Cheng X, Du DHC (2008). Real-time detection of clone attacks in wireless sensor networks. In Distributed Computing Systems, 2008. ICDCS'08. The 28th International Conference on (pp. 3–10). IEEE.

[13] Xing K, Cheng X, Ma L, Liang Q (2007). Superimposed code based channel assignment in multi-radio multi-channel wireless mesh networks. In Proceedings of the 13th annual ACM international conference on Mobile computing and networking (pp. 15–26). ACM.

[14] Znaidi W, Minier M, Ubéda S (2009). Hierarchical node replication attacks detection in wireless sensors networks. In Personal, Indoor and Mobile Radio Communications, 2009 IEEE 20th International Symposium on (pp. 82–86). IEEE.

[15] Yu CM, Lu CS, Kuo, SY (2012). CSI: compressed sensing-based clone identification in sensor networks. In Pervasive Computing and Communications Workshops (PERCOM Workshops), 2012 IEEE International Conference on (pp. 290–295). IEEE.

[16] Conti M, Di Pietro R, Mancini LV, Mei A (2007). A randomized, efficient, and distributed protocol for the detection of node replication attacks in wireless sensor networks. In Proceedings of the 8th ACM international symposium on Mobile ad hoc networking and computing (pp. 80–89). ACM.

[17] Conti M, Di Pietro R, Mancini LV, Mei A (2011). Distributed detection of clone attacks in wireless sensor networks. Dependable and Secure Computing, IEEE Transactions on, 8(5), 685–698.

[18] Bekara C, Laurent-Maknavicius M (2007). A new protocol for securing wireless sensor networks against nodes replication attacks. In Wireless and Mobile Computing, Networking and Communications, 2007. WiMOB 2007. Third IEEE International Conference on (pp. 59–59). IEEE.

[19] Bekara C, Laurent-Maknavicius M (2012). Defending against nodes replication attacks on wireless sensor networks.

[20] Ko LC, Chen HY, Lin GR (2009). A neighbor-based detection scheme for wireless sensor networks against node replication attacks. In Ultra Modern Telecommunications & Workshops, 2009. ICUMT'09. International Conference on (pp. 1–6). IEEE.

[21] Ho JW (2010). Distributed detection of node capture attacks in wireless sensor networks. INTECH Open Access Publisher.

[22] Ho JW, Liu D, Wright M, Das SK (2009). Distributed detection of replica node attacks with group deployment knowledge in wireless sensor networks. Ad Hoc Networks, 7(8), 1476–1488.

[23] Sei Y, Honiden S (2008). Distributed detection of node replication attacks resilient to many compromised nodes in wireless sensor networks. In Proceedings of the 4th Annual International Conference on Wireless Internet (p. 28). ICST (Institute for Computer Sciences, Social-Informatics and Telecommunications Engineering).

[24] Parno B, Perrig A, Gligor V (2005). Distributed detection of node replication attacks in sensor networks. In Security and Privacy, 2005 IEEE Symposium on (pp. 49–63). IEEE.

[25] Zhu B, Addada VGK, Setia S, Jajodia S, Roy S (2007). Efficient distributed detection of node replication attacks in sensor networks. In Computer Security Applications Conference, 2007. ACSAC 2007. Twenty-Third Annual (pp. 257–267). IEEE.

[26] Zhu B, Setia S, Jajodia S, Roy S, Wang L (2010). Localized multicast: efficient and distributed replica detection in large-scale sensor networks. Mobile Computing, IEEE Transactions on, 9(7), 913–926.

[27] Zhang M, Khanapure V, Chen S, Xiao X (2009). Memory efficient protocols for detecting node replication attacks in wireless sensor networks. In Network Protocols, 2009. ICNP 2009. 17th IEEE International Conference on (pp. 284–293). IEEE.

[28] Zeng Y, Cao J, Zhang S, Guo S, Xie L (2010). Random-walk based approach to detect clone attacks in wireless sensor networks. Selected Areas in Communications, IEEE Journal on, 28(5), 677–691.

[29] Khan WZ, Aalsalem MY, Saad NM, Xaing Y, Luan TH (2014). Detecting replicated nodes in Wireless Sensor Networks using random walks and network division. In Wireless Communications and Networking Conference (WCNC), 2014 IEEE (pp. 2623–2628). IEEE.

[30] Melchor C, Ait-Salem B, Gaborit P, Tamine K (2009) Active detection of node replication attacks. International Journal of Computer Science and Network Security, 2009; 9(2):13–21.

[31] Li Z, Gong G (2009). Randomly directed exploration: An efficient node clone detection protocol in wireless sensor networks. In Mobile Adhoc and Sensor Systems, 2009. MASS'09. IEEE 6th International Conference on (pp. 1030–1035). IEEE.

[32] Chano KIM, Seungjae SHIN, Chanil PARK (2009). A resilient and efficient replication attack detection scheme for wireless sensor networks. IEICE transactions on information and systems, 92(7), 1479–1483.

[33] Zhu C, Sun S, Wang L, Ding S, Wang J, Xia C (2014) Promotion of cooperation due to diversity of players in the spatial public goods game with increasing neighborhood size. Physica A: Statistical Mechanics and its Applications 406, 145–154. Online publication date: 1-Jul-2014.

[34] Xia C, Miao Q, Wang J, Ding S (2014) Evolution of cooperation in the traveler’s dilemma game on two coupled lattices. Applied Mathematics and Computation 246, 389–398. Online publication date: 1-Nov-2014.

[35] Wang L, Li X, Zhang YQ, Zhang Y, Zhang K (2011). Evolution of scaling emergence in large-scale spatial epidemic spreading. PloS one, 6(7), e21197 doi: 10.1371/journal.pone.0021197 21747932 10.1371/journal.pone.0021197 PMC3128583

[36] Zhang Y, Wang L, Zhang YQ, Li X (2012). Towards a temporal network analysis of interactive WiFi users. EPL (Europhysics Letters), 98(6), 68002.

[37] Wang L, Li X (2014). Spatial epidemiology of networked metapopulation: An overview. Chinese Science Bulletin, 59(28), 3511–3522. 10.1007/s11434-014-0499-8 PMC7088704 32214746

[38] Wang L, Wang Z, Zhang Y Li, X (2013). How human location-specific contact patterns impact spatial transmission between populations?. Scientific reports, 3. 10.1038/srep01468 PMC3601479 23511929

[39] Dutertre B, Cheung S, Levy J (2004). Lightweight key management in wireless sensor networks by leveraging initial trust. Technical Report SRI-SDL-04-02, SRI International.

[40] Ratnasamy S, Karp B, Yin L, Yu F, Estrin D, Govindan R, et al. (2002). GHT: a geographic hash table for data-centric storage. In Proceedings of the 1st ACM international workshop on Wireless sensor networks and applications (pp. 78–87). ACM.

[41] Khan WZ, Aalsalem MY, Saad MNBM, Xiang Y (2013). Detection and mitigation of node replication attacks in wireless sensor networks: a survey. International Journal of Distributed Sensor Networks, 2013.

[42] Zhu WT, Zhou J, Deng RH, Bao F (2012). Detecting node replication attacks in wireless sensor networks: a survey. Journal of Network and Computer Applications, 35(3), 1022–1034.

[43] Khan WZ, Saad MNBM, Aalsalem M. Y (2013). Scrutinising well-known countermeasures against clone node attack in mobile wireless sensor networks. International Journal of Grid and Utility Computing, 4(2), 119–127.

[44] Seshadri A, Perrig A, Van Doorn L, Khosla P (2004). Swatt: Software-based attestation for embedded devices. In Security and Privacy, 2004. Proceedings. 2004 IEEE Symposium on (pp. 272–282). IEEE.

[45] Aalsalem MY, Taheri J, Zomaya AY (2010). A framework for real time communication in sensor networks. In Computer Systems and Applications (AICCSA), 2010 IEEE/ACS International Conference on (pp. 1–7). IEEE.

[46] Karp B, Kung HT (2000). GPSR: Greedy perimeter stateless routing for wireless networks. In Proceedings of the 6th annual international conference on Mobile computing and networking (pp. 243–254). ACM.

[47] Shah R. C, Roy S, Jain S, Brunette W (2003). Data mules: Modeling and analysis of a three-tier architecture for sparse sensor networks. Ad Hoc Networks, 1(2), 215–233.

[48] Aldous D, Fill J (2002). Reversible Markov chains and random walks on graphs. [Online]. Available: http://stat-www.berkeley.edu/users/aldous/RWG/book.html

[49] Ellis R (2001). Torus hitting times from green’s functions.

[50] Menezes AJ, Van Oorschot PC, Vanstone SA (1996). Handbook of applied cryptography. CRC press.

[51] Zuniga M, Avin C, Hauswirth M (2010). Querying dynamic wireless sensor networks with non-revisiting random walks In Wireless Sensor Networks (pp. 49–64). Springer Berlin Heidelberg.

[52] Friedman R, Kliot G, Avin C (2010). Probabilistic quorum systems in wireless ad hoc networks. ACM Transactions on Computer Systems (TOCS), 28(3), 7.

[53] Estrin D, Govindan R, Heidemann J, Kumar S (1999). Next century challenges: Scalable coordination in sensor networks. In Proceedings of the 5th annual ACM/IEEE international conference on Mobile computing and networking (pp. 263–270). ACM.

[54] Liu A, Ning P (2008). TinyECC: A configurable library for elliptic curve cryptography in wireless sensor networks. In Information Processing in Sensor Networks, 2008. IPSN'08. International Conference on (pp. 245–256). IEEE.

